# *Malat1* deficiency prevents hypoxia-induced lung dysfunction by protecting the access to alveoli

**DOI:** 10.3389/fphys.2022.949378

**Published:** 2022-08-29

**Authors:** Sandrine Sallé-Lefort, Stéphanie Miard, Cyndi Henry, Christian Arias-Reyes, François Marcouiller, Marie-Josée Beaulieu, Sophie Aubin, Ariane Lechasseur, Éric Jubinville, David Marsolais, Mathieu C. Morissette, Vincent Joseph, Jorge Soliz, Ynuk Bossé, Frédéric Picard

**Affiliations:** ^1^ Institut universitaire de cardiologie et de pneumologie de Québec, Québec, QC, Canada; ^2^ Faculty of Pharmacy, Université Laval, Quebec, QC, Canada; ^3^ Faculty of Medicine, Université Laval, Quebec, QC, Canada

**Keywords:** lung, respiration, alveolar volume, methacholine, elastance, resistance, hysteresivity, lncRNA

## Abstract

Hypoxia is common in lung diseases and a potent stimulator of the long non-coding RNA *Metastasis-Associated Lung Adenocarcinoma Transcript 1* (*MALAT1*). Herein, we investigated the impact of *Malat1* on hypoxia-induced lung dysfunction in mice. *Malat1*-deficient mice and their wild-type littermates were tested after 8 days of normoxia or hypoxia (10% oxygen). Hypoxia decreased elastance of the lung by increasing lung volume and caused *in vivo* hyperresponsiveness to methacholine without altering the contraction of airway smooth muscle. *Malat1* deficiency also modestly decreased lung elastance but only when tested at low lung volumes and without altering lung volume and airway smooth muscle contraction. The *in vivo* responsiveness to methacholine was also attenuated by *Malat1* deficiency, at least when elastance, a readout sensitive to small airway closure, was used to assess the response. More impressively, *in vivo* hyperresponsiveness to methacholine caused by hypoxia was virtually absent in *Malat1*-deficient mice, especially when hysteresivity, a readout sensitive to small airway narrowing heterogeneity, was used to assess the response. *Malat1* deficiency also increased the coefficient of oxygen extraction and decreased ventilation in conscious mice, suggesting improvements in gas exchange and in clinical signs of respiratory distress during natural breathing. Combined with a lower elastance at low lung volumes at baseline, as well as a decreased propensity for small airway closure and narrowing heterogeneity during a methacholine challenge, these findings represent compelling evidence suggesting that the lack of *Malat1* protects the access to alveoli for air entering the lung.

## Introduction

Hypoxia is a worrying feature of common lung diseases such as chronic obstructive pulmonary disease (COPD) (Pierson, 2000; Kent et al., 2011). While molecular and cellular mechanisms involved in lung diseases include a coordinated transcriptional reprogramming of several genes responsive to hypoxia (Choudhry and Harris, 2018) or hypoxemia (Tuder et al., 2007), extensive translational work is still required to determine the extent by which any alteration in these specific genes ultimately contributes to clinical symptoms.

One gene of growing interest is *Metastasis-associated lung adenocarcinoma transcript-1* (*MALAT1*, also called *NEAT2*), a highly conserved long non-coding RNA (lncRNA), which affects gene expression in parts by sponging miRNA (Tripathi et al., 2010; Eissmann et al., 2012; Nakagawa et al., 2012; Zhang et al., 2012; Gutschner et al., 2013; Tripathi et al., 2013; Michalik et al., 2014; West et al., 2014; Gast et al., 2016; Xiang et al., 2016; Li et al., 2017; Carter et al., 2018; Kim et al., 2018; Lei et al., 2018). *MALAT1* is cleaved at its 3′ end, which generates a small fragment of 58 base pairs, called *MALAT1-associated small cytoplasmic RNA* (*MASCRNA*) (Ma et al., 2015) that is exported to the cytoplasm. Although ubiquitously expressed, *MALAT1* is abundant in the normal lung (Ji et al., 2003), suggesting a possible involvement in the normal biology of this organ, although no role has been defined yet. Moreover, our group demonstrated that *MALAT1* transcription is dependent on hypoxia-triggered signalling pathways including hypoxia-inducible factor-1 (HIF-1)α (Salle-Lefort et al., 2016), which has been suggested to contribute to ventilatory acclimatization to hypoxia (Kline et al., 2002; Moya et al., 2020). Thus, we hypothesized that *MALAT1* could be involved in pulmonary adaptations to hypoxia and, thereby, could impact lung diseases deleteriously or beneficially by shaping the altered lung function caused by hypoxia.

The purpose of this study was to investigate the role of *Malat1* in lung dysfunction caused by hypoxia in mice. To this end, wild-type (Malat1^+/+^) and *Malat1*-deficient mice (Malat1^−/−^) were exposed for 8 days to either 21% ambient oxygen (O_2_) or 10% ambient O_2_, hereinafter called normoxia and hypoxia, respectively.

## Materials and methods

### Mice

Male mice with germline deletion of *Malat1* were generated as previously described (Miard et al., 2017). They were housed on a 12:12 light-dark cycle and were provided food and water *ad libitum*. Nine to 11-week-old males from independent litters were used for the experiments. They were tested after 8 days of exposure to 21% ambient O_2_ (normoxia) or 10% ambient O_2_ (hypoxia) using a hypoxic housing chamber continuously monitored with an Oxycycler system (BioSpherix, NY). All experiments were approved by the institutional animal care committee of Université Laval.

### RNA fluorescent *in situ* hybridization

Fluorescent *in situ* hybridization (FISH) experiments were performed on 4 mice per group to confirm the lack of *Malat1* in cell nuclei of Malat^−/−^ mice and to qualitatively evaluate the effect of hypoxia on *Malat1* expression in Malat^+/+^ mice. Mice were anesthetized and then perfused through the heart with sterile saline solution until the liver was translucent. The lung was subsequently excised and fixed by perfusing formalin (10%, containing 4% paraformaldehyde) for 5 min. The fixed lung was embedded with OCT and cut in 10 μm-thick sections. Stellaris® FISH probes, consisting of a set of mixed oligonucleotides recognizing mouse *Malat1* (NCBI gene ID:72289, NR_002847.2, from nucleotides 751 to 6982) and labelled with QASAR 570 Dye (SMF-3008-1, Biosearch technologies, Inc., Petaluma, CA, United States), were hybridized as recommended by the manufacturer onto at least two lung sections per mouse. Nuclei were stained with Hoescht (Sigma-Aldrich, ON, Canada) in Fluoromount G medium (Electron Microscopy Sciences, Hatfield, PA, United States). Imaging was performed on a Zeiss Observer Z1 (objective 63X with oil) coupled with LSM 800 lasers system (Zeiss, ON, Canada) and analyzed with Zen Blue software (Zeiss, ON, Canada). Fiji ImageJ software was used to quantify RNA FISH signals on a total of 3–5 pictures per animal.

### Respiratory mechanics

Respiratory mechanics was measured with the flexiVent (FX Module 1, SCIREQ, software v7.2.2, QC, Canada) as previously described (Mcgovern et al., 2013). Mice were anesthetized with ketamine and xylazine at 100 and 10 mg/kg, respectively. They were then tracheotomized and connected to the flexiVent with an 18-gauge metal cannula in a supine position. The mechanical ventilation was set at 150 breaths per min at a tidal volume of 10 ml/kg with an inspiratory-to-expiratory ratio of 2:3 and a positive end-expiratory pressure (PEEP) of 3 cmH_2_O. Once the ventilation was established, mice were paralyzed with 0.1 mg/kg of pancuronium bromide injected intramuscularly.

At the beginning of the experiments, two deep inflations to 30 cmH_2_O were performed for lung volume recruitment. The second deep inflation was used to quantify inspiratory capacity (IC). Following a brief period of stabilization, a partial pressure-volume (P-V) maneuver was also performed as described (Robichaud et al., 2017). The P-V maneuver consisted of inflating the lung by step increases of pressure from 3 to 30 cmH_2_O and then deflating it by step decreases back to 3 cmH_2_O. The changes in (quasi-static) volume at the different holding pressures were then plotted for both the inflating and deflating limbs of the maneuver to form the P-V loop. The deflating limb was analyzed using the Salazar-Knowles’s equation: V = A–B*e* ^−KP^, where V is volume, A is the asymptote on the volume-axis (it is a fair estimate of IC but assessed in quasi-static conditions), B is the difference between A and the extrapolated volume at which pressure would cross zero, K is an exponent describing the curvature of the upper portion of the deflation limb of the P-V loop, and P is pressure. The parameter K is especially useful, since it is a volume-independent indicator of lung tissue compliance (Boucher et al., 2022). Two additional parameters were calculated from the quasi-static P-V loop, namely quasi-static elastance (Est) and hysteresis (i.e., the area within the P-V loop).

Two other volume perturbation maneuvers, called the Snapshot-150 and the Quick Prime-3, were also performed at baseline. In contrast to maneuvers described above, these are maneuvers of small amplitudes. More precisely, the Snapshot-150 and the Quick Prime-3 impose swings in volume approximately equivalent and below the range of tidal volume, respectively. They were actuated twice in an alternating fashion, each being intercalated by 8 s of tidal breathing to prevent desaturation. The volume perturbation imposed by the Snapshot-150 is a single sine wave (2.5 Hz) that allows resistance (Rrs) and elastance (Ers) of the respiratory system to be calculated based on the linear single-compartment model (Bates, 2009). The volume perturbation imposed by the Quick Prime-3 is a composite flow signal made of 13 sine waves of mutually prime frequencies with different amplitudes and phases, that allows Newtonian resistance (R_N_), tissue resistance (G) and tissue elastance (H) to be calculated based on the constant phase model (Hantos et al., 1992). Hysteresivity (η) was then calculated from the ratio of G over H.

After baseline measurements, mice were challenged with methacholine. The challenge was performed by nebulizing methacholine directly into the endotracheal cannula at incremental concentrations. For each concentration, the nebulizer (Aeroneb Lab, Aerogen Inc, Ireland) was operating for a duration of 10 s at a duty cycle of 50% during regular ventilation. The concentrations were nebulized at 5-min intervals in the following order: saline, 1, 3, 10, 30 and 100 mg/ml. The response was monitored after each concentration using the Snapshot-150 and the Quick Prime-3. Each of these volume perturbation maneuvers was actuated 10 times in an alternating fashion after each methacholine concentration, starting 10 s after the end of nebulization. Again, 8 s of tidal breathing was intercalated between each maneuver. For all readouts (Rrs, Ers, R_N_, G, H and η), the peak value after each concentration was used to assess the response to methacholine. The linear two-point concentration-response slope was then calculated to assess the degree of airway responsiveness in each mouse, as previously described (O’connor et al., 1987). Briefly, for each readout, the maximal change caused by methacholine from the value after the nebulization of saline was divided by the final concentration of methacholine (i.e., 100 mg/ml).

### Tissue collection

Mice were euthanized with ketamine and xylazine at 200 and 10 mg/ml, respectively. Several tissues were then collected, including blood, bronchoalveolar lavages, the left lung and the trachea. Lobes of the right lung were also collected and flash-frozen in liquid nitrogen and stored at −80°C until used for protein extraction.

### Circulating levels of erythropoietin

Mice were exsanguinated and the collected blood was treated with an anticoagulant and centrifuged to isolate the plasma. The samples were preserved at −80°C until processed to quantify plasma erythropoietin by ELISA (R&D systems Quantikine ELISA Mouse Erythropoietin immunoassay MEP00B).

### Inflammation in bronchoalveolar lavages

The bronchoalveolar lavages were collected by three repeated injections and aspirations of 1 ml of sterile PBS. The total aspirated volume was centrifuged at 500 g for 5 min. The recovered pellet was resuspended to count total cells and to prepare cytospins. The latter were stained with a modified May-Grünwald Giemsa (HemaStain Set, Fisher Scientific, Kalamazoo, MI, United States) to count macrophages, lymphocytes, neutrophils and eosinophils.

### Immunohistochemistry to quantify the content of smooth muscle within the airway wall

Exploratory experiments were performed in four mice per group to qualitatively evaluate the content of smooth muscle within the airway wall. After collection, the left lung was perfused with 4% paraformaldehyde, fixed at 4°C for 3 days, embedded in paraffin, cut in 5 μm-thick sections and mounted on glass slides. Paraffin was then removed by 2 washes in Toluene for 5 min each. Then, tissue was rehydrated by successive baths in 100%, 95%, 70% and 50% of ethanol for 3 min and then washed in dH_2_O. Antigen retrieval was performed by bathing slides in citrate buffer (pH 6) for 12 min at 95°C and then left bathing until the buffer reached room temperature. Slides were then washed in PBS and blocked for 1 h at room temperature in blocking buffer (1% BSA, 5% Normal Goat Serum, 0.3% Triton 100x in PBS). Slides were incubated overnight at 4°C with anti α -SMA (Abcam #ab5694) diluted 1:100 in PBS containing 1% BSA, 1% Normal Goat Serum, 0.3% Triton 100x. Staining was then visualized by using the anti-rabbit HRP-DAB staining kit from R&D system (CTS005) according to the manufacturer’s protocol. The images were converted into high-resolution digital data using the NanoZoomer Digital scanner (Hamamastu Photonics, Bridgewater, United States). Three to five lung sections per mouse were analyzed.

### Western blot analysis to quantify the content of airway smooth muscle within the lung

Pieces of lung tissues were weighted and minced with a mechanical disruptor in a lysis buffer (50 mM HEPES-KOH pH 7.4, 40 mM NaCl, 2 mM EDTA, 1.5 mM NaVO4, 50 mM NaF, 10 mM NaPyrophosphate, 10 mM NaBetaGlycerophosphate, 1% Triton x-100 and 1:1000 Protease Inhibitor Cocktail). The preparations were then incubated 10 min on ice and centrifuged for 10 min at 11000 g at 4°C to collect the supernatants. Protein concentrations in the supernatants were determined as previously described (Salle-Lefort et al., 2016). Thirty μg of proteins were loaded onto 8% SDS-polyacrylamide gels and separated until the loading blue went out of the gel. Separated proteins were transferred to PVDF membranes according to the Trans-Blot Turbo Transfer System manual from Bio-Rad. The membranes were then blocked for 1 hour at room temperature in TBS (5 mM Tris-HCl pH 7.5, 15 mM NaCl, 0.02% Tween 20, and 0.04% NP40)-5% milk and then blotted overnight at 4°C with agitation with the following primary antibodies: a rabbit anti-α-SMA (Abcam #ab5694, diluted 1:1000) or a mouse anti-β-actin (Millipore, MAB1501, diluted 1:10000) in TBS-5% milk. Membranes were then washed three times in TBS and incubated 1 h at room temperature with anti-rabbit or anti-mouse immunoglobulin G, as appropriate, conjugated to HRP. After three washes of 8 min, signals were detected with ECL™ Western Blotting Detection Reagents (GE Healthcare) on Kodak films.

### Contractile capacity of airway smooth muscle

The contractile capacity of airway smooth muscle was assessed *ex vivo* on excised tracheas, as previously described (Mailhot-Larouche et al., 2018). The whole tracheas was mounted in a thermostatic organ bath filled with Krebs solution kept at 37°C. More precisely, the trachea was held horizontally between two stainless steel triangles with one side of each running into the tracheal lumen. The lower triangle was attached to a hook at the bottom of the bath and the other one was connected by a surgical thread to a force transducer (Harvard Apparatus, St-Laurent, Canada). The latter measures isometric force. A distending force of 5 mN was applied at baseline. Prior to any recording, the trachea was subjected to a period of conditioning, during which time the airway smooth muscle was stimulated to contract repeatedly for 5 min at 10-min intervals with 10^−5^ M of methacholine until a reproducible force was recorded. The trachea was then challenged with two spasmogens successively in a randomized order: 1-methacholine, which causes contraction via a receptor-mediated mechanism; and 2- potassium chloride (KCl), which causes contraction by depolarizing the plasmalemma and, therefore, via a mechanism independent of a cell-surface receptor. The concentrations of each spasmogen were added serially at 5-min intervals in a cumulative fashion; in log increments from 10^−8^ to 10^−4^ M for methacholine and in doubling concentrations from 20 to 160 mM for KCl. The trachea was washed extensively after the first spasmogen. The test with the second spasmogen started after a period of at least 30 min following the return to baseline force. The peak value of force recorded at each concentration was used to assess the degree of responsiveness.

The ability of airway smooth muscle to relax was also evaluated. For these experiments, the trachea was pre-contracted with the EC_50_ of methacholine (i.e., the concentration causing 50% of the maximal response). Once the force plateau was achieved, incremental concentrations of isoproterenol were added serially into the organ bath in a cumulative fashion: in log increments from 10^−7^ to 10^−5^ M at 5-min intervals. The minimal value of force recorded at each concentration, expressed in raw value or in percentage from the force generated by the EC_50_ of methacholine, was used to assess the degree of responsiveness to isoproterenol.

### Whole-body plethysmography

Mice were individually placed in a whole-body flow-through plethysmograph chamber (EMKA Technologies, Paris, France). For recordings, mice exposed for 8 days to normoxia or hypoxia were placed under similar conditions (normoxia or hypoxia, respectively) for 3 h. The chamber was constantly flushed with fresh air or the hypoxic gas (200 ml/min) and a sub-sampling pump (75 ml/min) was used to collect outflowing air. A built-in pneumotachograph measured the airflow in and out of the chamber related to breathing. Before the experiments, airflow was calibrated by rapidly injecting 0.5 ml of air within the chamber. Water pressure, CO_2_, and O_2_ levels in the outflowing and inflowing air were continuously monitored using specific gas analyzers (RH-300, CA-10, and FC-10; Sable Systems, Las Vegas, United States), previously calibrated using a certified dry gas mix (Linde Canada, QC, Canada). The temperature of the plethysmograph chamber was recorded with a thermocouple. Rectal temperature was measured at the end of the recording session (Ganouna-Cohen et al., 2022). All signals were digitized (Micro 1401 data acquisition system; Cambridge Electronic Design, Cambridge, England), stored and analyzed on a computer using the Spike 2 software (version 7.06—CED). Breathing frequency was measured from the recordings, and tidal volume was calculated using the standard equation for whole-body plethysmography (Boukari et al., 2016), with rectal temperature taken into account. Minute ventilation (VE) was calculated as breathing frequency x tidal volume. Water pressure, CO_2_, and O_2_ levels in inflowing and outflowing gases were used to calculate O_2_ consumption and CO_2_ production rate as previously described (Boukari et al., 2016).

### Statistical analysis

Data shown are means ± SEM. Two-way ANOVAs were used to assess the effect of hypoxia, *Malat1* deficiency and their interaction. When interactions between main factors were statistically significant, Tukey post-hoc tests were performed. Prism v.8.01 (GraphPad Software, San Diego, United States) was used for all statistical analyses. Differences between groups were considered statistically significant at *p* ≤ 0.05.

## Results

### *Malat1* expression

**FIGURE 1:**
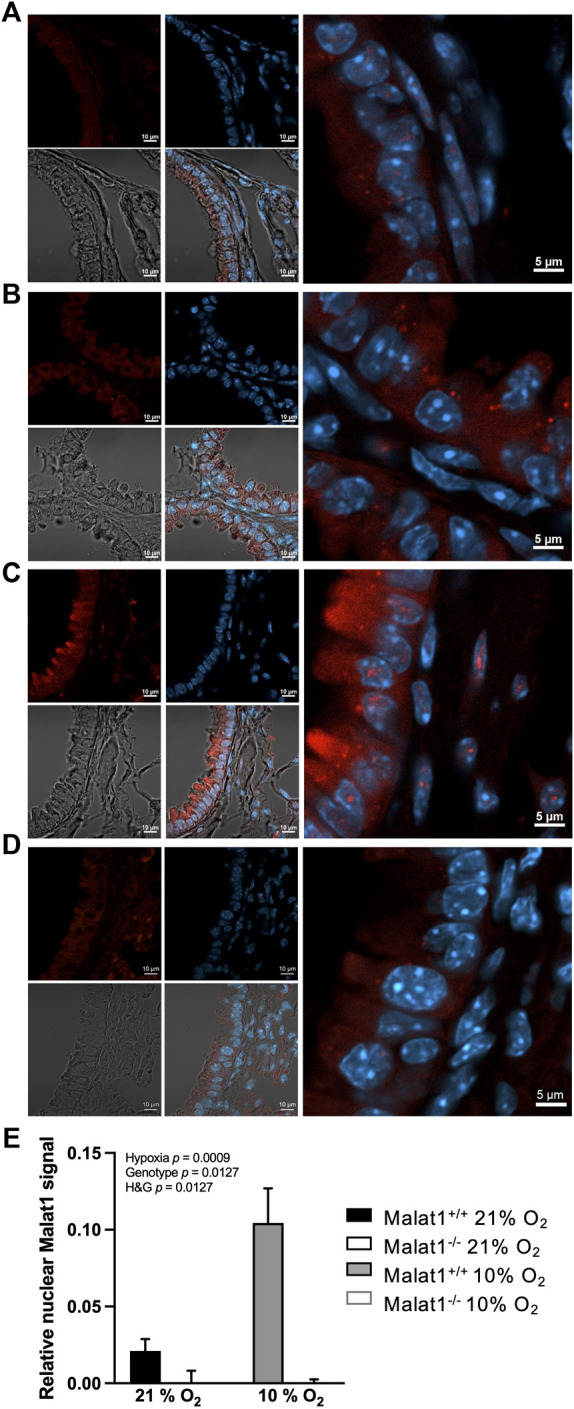
*Malat1* expression. Representative confocal images of RNA FISH performed on lung sections from Malat1^+/+^ mice housed at 21% O_2_ **(A)**, Malat1^−/−^ mice housed at 21% O_2_ **(B)**, Malat1^+/+^ mice housed at 10% O_2_ **(C)**, and Malat1^−/−^ mice housed at 10% O_2_ **(D)**. Tissue sections derived from four independent mice per group were examined and one representative image per group is shown. *Malat1* appears in red and Hoescht-stained nuclei appear blue. 3–5 lung slices per animal were analyzed and used for quantification **(E)**.

Exposure to hypoxia increased circulating levels of erythropoietin in both Malat1^+/+^ (109 ± 16 in normoxia (*n* = 8) *vs.* 224 ± 60 pg/ml in hypoxia (*n* = 11), *p* = 0.0008, mean ± SEM) and Malat1^−/−^ mice (119 ± 19 in normoxia (*n* = 8) *vs.* 203 ± 47 pg/ml in hypoxia (*n* = 10), *p* = 0.02), confirming normal systemic sensing. Hypoxia triggered a mild inflammation, characterized by an approximately 4-fold increase in macrophages in bronchoalveolar lavages, with small changes in lymphocytes, eosinophils and neutrophils (data not shown). The deficiency in *Malat1* did not modify inflammation (data not shown). RNA-FISH analyses showed that *Malat1* expression in the nucleus increased approximately 5-fold upon hypoxia in wild-type mice (Figures 1A,C,E) but was absent in those of *Malat1*-deficient animals (Figures 1B,D,E).

### Respiratory mechanics

**FIGURE 2:**
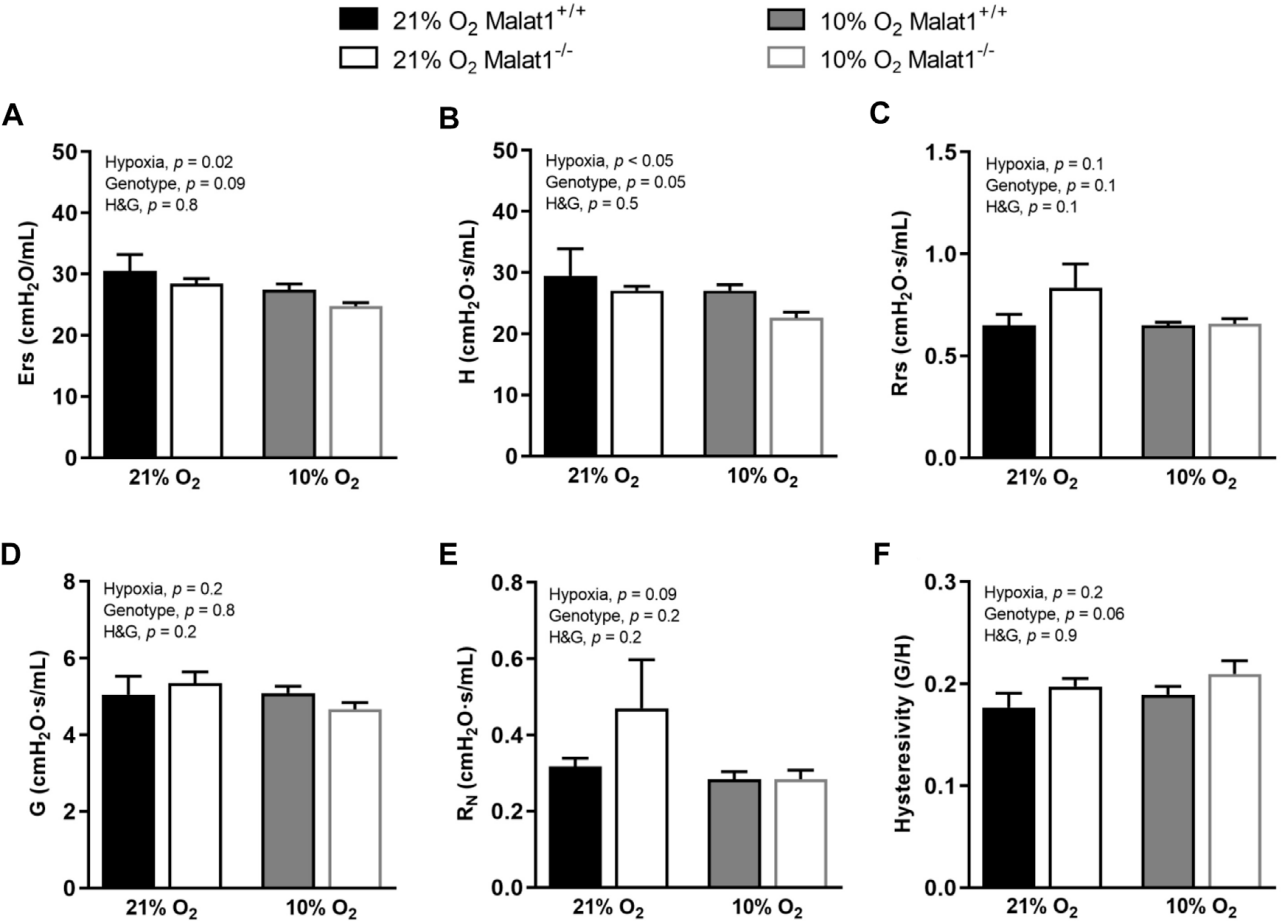
*In vivo* respiratory mechanics at baseline. **(A)** Respiratory system elastance (Ers), **(B)** tissue elastance (H), **(C)** respiratory system resistance (Rrs), **(D)** tissue resistance (G), (E) Newtonian resistance (R_N_) and **(F)** hysteresivity (G/H = η) in Malat1^+/+^ and Malat1^−/−^ mice housed in normoxia or hypoxia for 8 days. *n* = 5, 8, 9 and 8 for Malat1^+/+^ in normoxia, Malat1^−/−^ in normoxia, Malat1^+/+^ in hypoxia and Malat1^−/−^ in hypoxia groups, respectively. Data were analyzed by two-way ANOVA. *p = 0.05, **p = 0.001, ***p < 0.0001.

**FIGURE 3:**
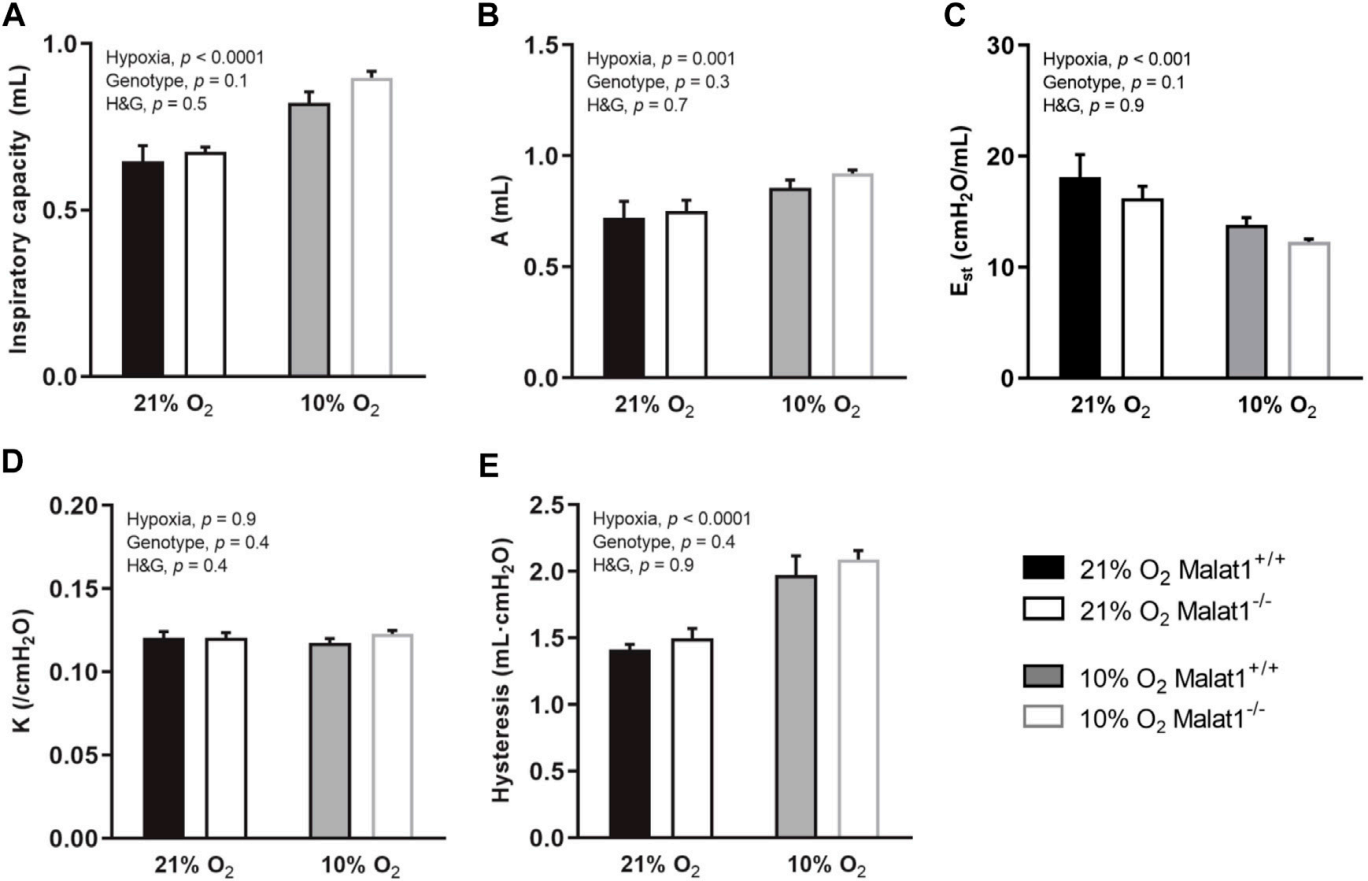
*In vivo* respiratory mechanics measured over a large range of lung volumes at baseline. Inspiratory capacity **(A)**; the parameter A of Salazar-Knowles’ equation **(B)**; quasi-static elastance (Est) **(C)**, the parameter K of Salazar-Knowles’ equation **(D)**; and hysteresis **(E)** in Malat1^+/+^ and Malat1^−/−^ mice housed in normoxia or hypoxia for 8 days. *n* = 8, 8, 11 and 10 for Malat1^+/+^ in normoxia, Malat1^−/−^ in normoxia, Malat1^+/+^ in hypoxia and Malat1^−/−^ in hypoxia groups, respectively. Data were analyzed by two-way ANOVA.

Respiratory system elastance (Ers), tissue elastance (H), respiratory system resistance (Rrs), tissue resistance (G), Newtonian resistance (R_N_) and hysteresivity (η), which is G/H, were measured at baseline (Figure 2). Hypoxia decreased Ers and H in both genotypes (Figures 2A,B), but did not affect Rrs, G, R_N_ and η (Figures 2C–F). *Malat1* deficiency also decreased H (Figure 2B), but had no significant effect on Ers, Rrs, G, R_N_ and η (Figures 2A,C–F). Other variables were measured using maneuvers that probe the lung over a larger range of volumes, including a deep inflation maneuver and the partial quasi-static pressure-volume maneuver. Hypoxia increased the inspiratory capacity (Figure 3A), the parameter A of the Salazar-Knowles’ equation (Figure 3B), and hysteresis (Figure 3E). It also decreased the lung quasi-static elastance (Est) (Figure 3C). However, the parameter K of the Salazar-Knowles’ equation, a volume-independent indicator of lung tissue compliance, was not affected by hypoxia (Figure 3D). This suggests that the increased compliance caused by hypoxia was chiefly due to an increased lung volume, and not by altering the mechanical properties of the lung tissue. *Malat1* deficiency had no effect on any of the variables measured over a large range of lung volumes (Figure 3), suggesting that its effect on elastance (H) was only apparent when it was measured over a small range of lung volumes.

### *In vivo* responsiveness to methacholine

**FIGURE 4:**
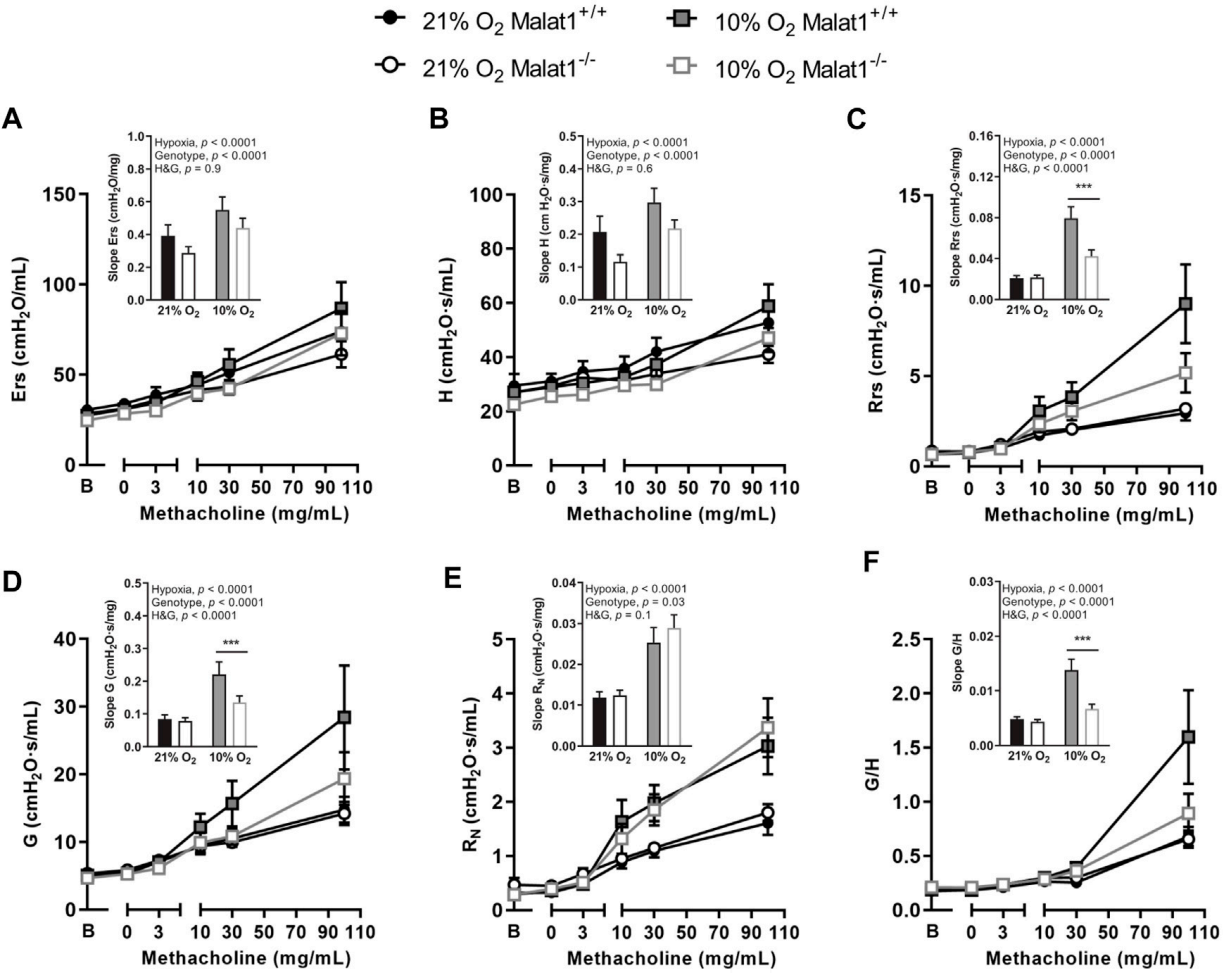
Responsiveness to methacholine *In vivo* respiratory mechanics in response to incremental concentrations of nebulized methacholine in Malat1^+/+^ and Malat1^−/−^ male mice housed at either 21% O_2_ or 10% O_2_ for 8 days. The bar graph in the inset presents the slope of the methacholine concentration-response curve in each group. The readouts used to assess the degree of responsiveness to methacholine were: Ers, elastance of the respiratory system **(A)**; H, lung tissue elastance **(B)**; Rrs, resistance of the respiratory system **(C)**; G, lung tissue resistance **(D)**; R_N_, Newtonian resistance to airflow in the large conducting airways **(E)**; and η, hysteresivity **(F)**, which is G/H. *n* = 8, 8, 11 and 10 for Malat1^+/+^ in normoxia, Malat1^−/−^ in normoxia, Malat1^+/+^ in hypoxia and Malat1^−/−^ in hypoxia groups, respectively. Data were analyzed by two-way ANOVA and Tukey post-hoc tests. ***p < 0.0001.

Mice were then challenged with nebulized methacholine. Hypoxia increased gains in Ers, H, Rrs, G, R_N_ and η induced by methacholine (Figure 4), confirming hyperresponsiveness. Gains in Ers and H induced by methacholine were attenuated in Malat1^−/−^ mice (Figures 4A,B). In contradistinction, the gain in R_N_ caused by methacholine was slightly increased by *Malat1* deficiency (Figure 4E). More impressively, there were significant and very strong interactions between the ambient O_2_ concentration (normoxia vs. hypoxia) and the genotype (Malat1^−/−^ *vs.* Malat1^+/+^) for gains in Rrs (Figure 4C), G (Figure 4D), and η (Figure 4F) induced by methacholine. While the deficiency in *Malat1* had no effect on methacholine-induced gains in Rrs, G, and η (Figures 4C,D,F) in normoxia, it virtually abrogated the excessive response to methacholine caused by hypoxia (Figures 4C,D,F). The most prominent readout to detect this protective effect of *Malat1* deficiency on airway hyperresponsiveness was hysteresivity (η).

### Airway smooth muscle structure and function

**FIGURE 5:**
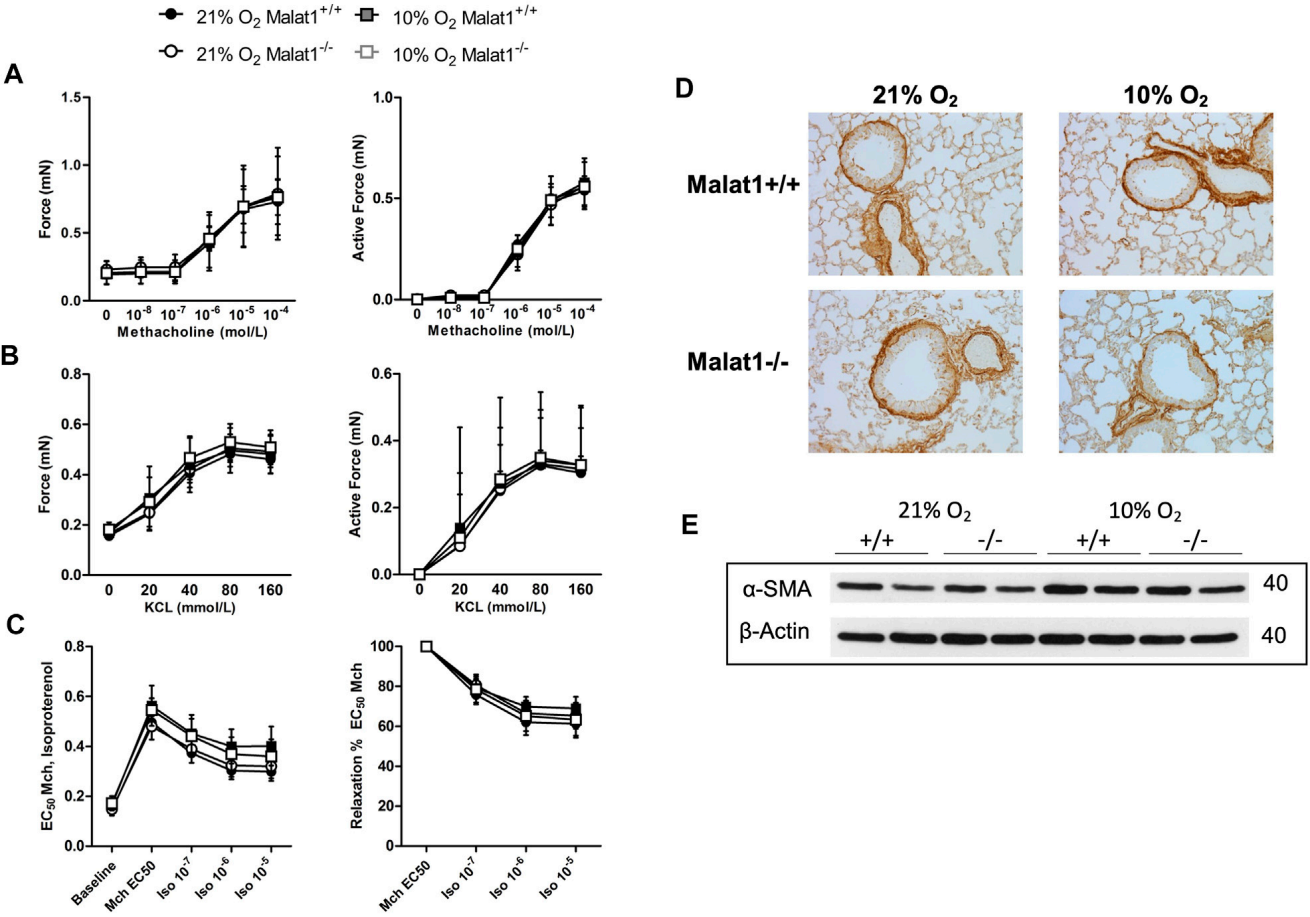
Airway smooth muscle The contractile capacity of airway smooth muscle was assessed on excised tracheas derived from Malat1^+/+^ and Malat1^−/−^ mice housed in normoxia or hypoxia for 8 days. The contraction elicited by incremental concentrations of either methacholine **(A)** or potassium chloride (KCl) **(B)**, as well as the relaxation elicited by incremental concentrations of isoproterenol on tracheas pre-contracted with the EC_50_ of methacholine **(C)**, were assessed. *n* = 6/group. No differences were detected at any concentrations based on two-way ANOVAs. The content of smooth muscle within the airway wall was also assessed qualitatively following immunohistochemical staining of α-smooth muscle actin (α-SMA) on lung slices **(D)**. Three to five slices per mouse from four animals per group were examined and one representative image per group is shown. There was no significant effect of genotype or hypoxia. The content of smooth muscle was also assessed quantitatively by measuring the expression of α-SMA by western blot in tissue homogenates of the lung of 2 mice per group **(E)**, again observing no significant effect of genotype or hypoxia.

The capacity of airway smooth muscle to contract and to relax was also tested directly on excised tracheas (Figures 5A–C and Supplementary Material). There were no differences between groups. The content of smooth muscle, both within the airway wall and within the lung, was also not affected by hypoxia, *Malat1* deficiency or their combination (Figures 5D,E).

### Ventilation and gas exchange

**FIGURE 6:**
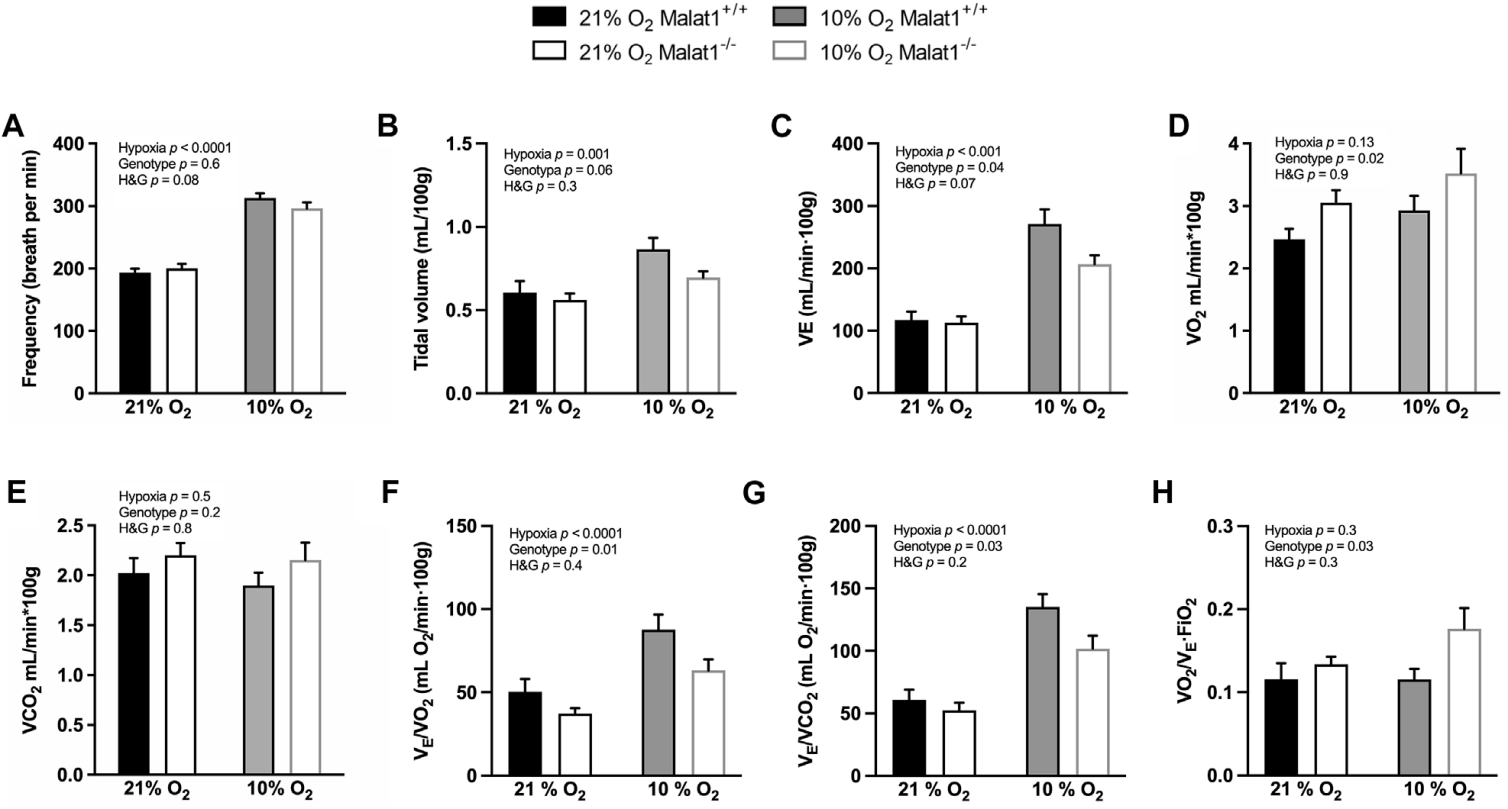
Ventilation and oxygen extraction. Malat1^+/+^ and Malat1^−/−^ male mice were recorded in a whole-body plethysmograph under normoxia (21% O_2_) after 8 days of normoxia (21% O_2_) or under hypoxia (10% O_2_) after 8 days of hypoxia (10% O_2_): The variables measured were: frequency of breathing **(A)**, tidal volume **(B)**, minute ventilation **(C)**, VO_2_ **(D)**, VCO_2_ **(E)**, ventilatory equivalent of oxygen **(F)**, ventilatory equivalent of carbon dioxide **(G)**, and oxygen extraction coefficient **(H)**. *n* = 9 per group. Data were analyzed by two-way ANOVA.

Supplemental experiments were conducted in the whole-body plethysmograph to measure the pattern of breathing and gas exchange in conscious mice. Hypoxia significantly decreased rectal temperature (*p* < 0.0001) but there was no difference between genotypes (*p* = 0.8) in normoxia or hypoxia (data not shown). Hypoxia significantly increased breathing frequency, tidal volume and ventilation (VE) (Figures 6A–C). Compared to Malat1^+/+^ mice, Malat1^−/−^ mice significantly consumed more O_2_ (*p* = 0.02) but produced similar amounts of CO_2_ as measured by whole-body plethysmography, with no statistical interaction with hypoxia (Figures 6D,E). As expected from the lower ambient concentration of O_2_, hypoxia also increased the ratio of VE on O_2_ consumption (VE/VO_2_) and the ratio of VE on CO_2_ production rate (VE/VCO_2_) (Figures 6F,G). However, hypoxia had no effect on the coefficient of O_2_ extraction (Figure 6H, VO_2_/[VE x FiO_2_]: the ratio of O_2_ consumption over VE x the inhaled fraction of O_2_). Compared to Malat1^+/+^ mice, Malat1^−/−^ mice demonstrated decreases in VE (Figure 6C), VE/VO_2_ (Figure 6F) and VE/VCO_2_ (Figure 6G), as well as an increase in the coefficient of O_2_ extraction (Figure 6H). Together, these results suggest that while hypoxia increased ventilation without affecting the pulmonary O_2_ extraction, *Malat1* deficiency decreased ventilation by improving alveolar gas exchange.

## Discussion

Our study demonstrated that hypoxia elicits obvious lung dysfunction, including: 1- a decreased elastance (Ers, H & Est), chiefly due to an increase in lung volume (IC & A) and without altering the compliance of the lung tissue (i.e., hypoxia did not change K); and 2- a marked increase in the degree of *in vivo* responsiveness to methacholine without altering the structure and the function of the airway smooth muscle. The physiological effects of *Malat1* deficiency were modest prior to methacholine challenge. Yet, Malat1^−/−^ mice exhibited a decreased elastance compared to Malat1^+/+^ mice when it was measured over a low (H) but not a high (Est) range of lung volumes. When mice were challenged with methacholine, *Malat1* deficiency attenuated the gain in elastance, including Ers and H, which are two sensitive readouts to small airway closure. More impressively, the potentiating effect of hypoxia on the methacholine-induced increase in resistance (Rrs & G) was reduced by *Malat1* deficiency. In fact, hyperresponsiveness to methacholine caused by hypoxia was virtually abrogated by *Malat1* deficiency when hysteresivity (η), a readout sensitive to small airway narrowing heterogeneity, was used to assess the response. In conscious mice breathing naturally, hypoxia increased breathing frequency, tidal volume and ventilation, in addition to increase ventilation per units of O_2_ consumption and CO_2_ production (VE/VO_2_ and VE/VCO_2_). The deficiency in *Malat1* improved most of these latter readouts, in addition to enhance the coefficient of O_2_ extraction, indicating that the lack of *Malat1* attenuated clinical signs of respiratory distress by improving gas exchange.

### The access to alveolar volume

Apart from the intrinsic mechanical properties of the lung tissue, another extremely important determinant of *in vivo* elastance is alveolar volume. Since the lung was the same size between Malat1^+/+^ and Malat1^−/−^ mice, which was attested by lack of differences in IC and A, the decreased elastance (i.e., H) in Malat1^−/−^ mice may stem from a greater access to alveolar volume; *viz.*, the total alveoli reachable from the mouth. Indeed, it is well understood that derecruitment caused by either alveolar collapse (atelectasis) or small airway closure increases elastance (Lutchen and Gillis, 1997; Wagers et al., 2004; Farrow et al., 2012; Al-Alwan et al., 2014; Kaminsky et al., 2019). This is because derecruitment directs the oscillating airflow into a smaller portion of the lung, which is then subjected to relatively larger volume perturbations. As a result, greater changes in pressure for the same changes in volume entering the mouth are measured, the ensuing calculated elastance (pressure/volume) is increased, and the lung thus appears stiffer. Inversely, factors decreasing derecruitment would decrease *in vivo* elastance. We thus propose that the decreased elastance (H) in Malat1^−/−^ *versus* Malat1^+/+^ mice stemmed from a greater access to alveolar volume due to less derecruitment. This is especially relevant herein given that derecruitment affects the accessible alveolar volume at low but not at high lung volumes, explaining why the decreased elastance (H) caused by *Malat1* deficiency was only seen at low lung volumes. Together, these findings suggested that the lack of *Malat1* provides a greater access to alveoli for air entering the lung at low lung volumes.

### Responsiveness to methacholine

Stimulating the contraction of airway smooth muscle with methacholine is a useful means to detect subtle lung alterations. The results of the present study demonstrated that the deficiency in *Malat1* reduces the gain in elastance (Ers and H) induced by methacholine. This suggested that the deficiency in *Malat1* not only provides a greater access to alveoli at low lung volumes at baseline as stated above, but also protects the access to alveoli by preventing small airway closure during a methacholine challenge.

As expected, hypoxia clearly caused hyperresponsiveness to methacholine, irrespective of the readout used (Rrs, Ers, R_N_, G, H and η) to assess the response. Surprisingly, however, the enhancing effect of hypoxia on methacholine-induced gains in Rrs, G and η was much reduced in Malat1^−/−^ mice. The significance of this finding is described next.

First, it is essential to understand that derecruitment caused by pure airway closure does not change η (Bates et al., 2009), since both G and H are expected to increase by the same relative amount when parts of the lung are no longer reachable from the mouth. Second, it is important to know that, although G is purported to reflect tissue resistance, it is also sensitive to small airway narrowing heterogeneity (Lutchen et al., 1996). The gain in η caused by methacholine in the present study means that G had increased relatively more than H in every group. This is not atypical, since narrowing heterogeneity invariably precedes airway closure (Lutchen et al., 1996; Petak et al., 1997; Bellardine et al., 2005). Importantly, this methacholine-induced gain in η was markedly amplified by hypoxia, suggesting that the main effect of hypoxia was not necessarily to increase airway narrowing *per see*, but to increase narrowing heterogeneity. Again, this was not totally unexpected as small airway narrowing heterogeneity is one of the major causes of hyperresponsiveness in diseased lungs, as shown experimentally in animals (Lutchen et al., 1996; Petak et al., 1997; Wagers et al., 2004), as well as clinically in humans (King et al., 2004; Downie et al., 2007; Hardaker et al., 2011; Dame Carroll et al., 2015; Farrow et al., 2017). In the present study, narrowing heterogeneity may actually account, at least to a great extent, for the potentiation of methacholine-induced gain in Rrs caused by hypoxia, since Rrs embodies both airway resistance to airflow and G. Finally, the fact that the potentiating effect of hypoxia on methacholine-induced gains in Rrs, G and η was almost absent in Malat1^−/−^ mice indicates that the major effect of *Malat1* deficiency in pathologic conditions was to prevent airway narrowing heterogeneity, in addition to limit airway closure as outlined above.

Lastly, the slight increase in methacholine-induced narrowing of the large airways (R_N_) in Malat1^−/−^ *versus* Malat1^+/+^ mice can perhaps be ascribed to a smaller total lung impedance in Malat1^−/−^ mice due to less small airway narrowing heterogeneity and closure. This would enable more of the nebulized methacholine to penetrate the lung (Porra et al., 2018). Consequently, it would raise the activation level of the airway smooth muscle and its shortening, explaining why large airway narrowing was slightly but significantly greater in Malat1^−/−^ mice.

### Greater access to alveolar volume in conscious mice

An additional method was used to verify the conjecture stated above, about the greater access to alveoli conferred by *Malat1* deficiency at low lung volumes. We reasoned that this greater access to alveoli, in Malat1^−/−^ versus Malat1^+/+^ mice, should improve ventilation efficiency. This is because a greater number of alveoli should be ventilated in Malat1^−/−^ mice for any given tidal volume. By monitoring ventilation efficiency over a period of time, and assuming that Malat1 deficiency did not influence gas transport across the blood-gas barrier, subtle changes in accessibility to alveoli should thus be detected in mice breathing spontaneously at tidal volume. Consistent with this notion, *Malat1* deficiency significantly improved gas exchange, attested by a greater coefficient of O_2_ extraction. This supports, once again, that the access to alveolar volume was improved in Malat1^−/−^ mice at low lung volumes. Moreover, it suggested that the impact of *Malat1* deficiency in living mice was physiologically meaningful as it ultimately decreased ventilation.

## Conclusion

We conclude that the absence of *Malat1* seems to protect access to alveoli when the lung volume is about the tidal breathing range. This is suggested based on several evidence: 1- a lower elastance at low lung volumes; 2- a decrease in small airway closure, and also small airway narrowing heterogeneity in hypoxia-exposed mice, during a methacholine challenge; 3- an increase in the pulmonary O_2_ extraction during natural breathing; and 4- a resulting improvement in clinical signs (i.e., decreased ventilation). Altogether, the results support the notion that *Malat1* shapes rather detrimentally the altered lung function caused by hypoxia, and may perhaps play a similar role in lung disorders involving hypoxia, such as COPD.

## Data Availability

The raw data supporting the conclusions of this article will be made available by the authors, without undue reservation.
